# Stability of self-reported psychopathic traits in at-risk adolescents in youth welfare and juvenile justice institutions

**DOI:** 10.1186/s13034-022-00487-6

**Published:** 2022-06-28

**Authors:** H. Hachtel, N. Jenkel, K. Schmeck, M. Graf, J. M. Fegert, M. Schmid, C. Boonmann

**Affiliations:** 1 Department of Forensic Psychiatry, Psychiatric University Hospitals (UPK) Basel, Wilhelm Klein-Strasse 27, 4002 Basel, Switzerland; 2 Child and Adolescent Research Department, Psychiatric University Hospitals (UPK) Basel, Basel, Switzerland; 3 Department of Child and Adolescent Psychiatry and Psychotherapy, University Hospital of Ulm, Ulm, Germany; 4 Department of Psychology, University of Basel, Basel, Switzerland

**Keywords:** Psychopathic traits, YPI, Adolescents, Stability, Reliable change index, Residential care, Child welfare, Juvenile justice, Internalizing mental health problems, Externalizing mental health problems

## Abstract

**Background:**

The purpose of this study was to evaluate the self-reported stability of psychopathic traits in adolescents in residential care (both child welfare and juvenile justice placed juveniles) and potential influencing factors.

**Methods:**

We applied the Youth Psychopathic traits Inventory (YPI) in a sample of 162 adolescents (M = 15.0 years, SD = 1.3) over a mean time interval of 11 months (min. 6, max. 21 months, SD = 3.14).

**Results:**

There was no significant difference in YPI total score nor in the three underlying dimensions Grandiose-Manipulative (GM), Callous-Unemotional (CU), and Impulsive-Irresponsible (II) between t1 and t2. Furthermore, approximately 70% of the adolescents showed no clinically significant reliable change on the YPI total score (as measured with the reliable change index), 15% improved, 15% deteriorated. The strongest predictor for psychopathic traits at t2 were psychopathic traits at t1. Additional predictors for higher levels of general psychopathic traits was male sex, for CU-traits male sex and lower levels of internalizing mental health problems, and for II-traits higher levels of externalizing mental health problems. Generally, the three reliable change groups (increase, no change, decrease) did not seemed to differ on relevant factors.

**Conclusions:**

Our results add to the findings that psychopathic traits are relatively stable in this at-risk group over approximately a 1-year time interval. Research with a longer follow-up time and more time points is warranted to better interpret these results.

## Background

Psychopathic traits in children and adolescents are increasingly getting attention in both the scientific literature as well as clinical practice. These traits are often described using the three-dimensional conceptualization of Cooke and Michie [1], including the underlying dimensions Grandiose-Manipulative (GM), Callous-Unemotional (CU), and Impulsive-Irresponsible (II) traits, and tend to be associated with an earlier onset of delinquent behavior, higher levels of delinquent behavior, and higher rates of recidivism [2]. This interest in psychopathic traits is also reflected in the newly added limited prosocial emotion (LPE) specifier to the Conduct Disorder (CD) diagnosis in the Diagnostic and Statistical Manual (DSM)-5 [3], in order to better capture the small group of youth who are more likely to persist in antisocial behavior and might, therefore, be diagnosed with an antisocial personality disorder in adulthood. Nevertheless, there is little research regarding the stability of psychopathic traits in children, adolescents and young adults.

The stability of psychopathic traits is a long-standing discussion in the adults literature with opposing opinions on the efficacy of therapeutic interventions [4]. This is not surprising given the common belief that treatment might even have a negative effect on psychopathic traits [5]. A systematic review on the empirical evidence regarding untreatability of psychopathic characteristics in adults, however, reported that only one study suggested high psychopathic traits being associated with less favourable treatment outcomes and that individuals with higher levels of psychopathic traits could demonstrate similar therapeutic progress compared to others [6]. Other research suggested possible modest changes of psychopathic traits in the life course of adults even without therapeutic interventions [7].

These findings in adults may not inevitably be translated to minors, as adolescence is an important neuro-developmental phase including maturing and developing of the brain [8]. Literature suggests that increases in psychosocial maturity (among others responsibility, social perspective and temperance) are more pronounced in adolescents than in adults [9]. Hence, there is evidence that the transition from adolescence to young adulthood is marked by continuity of lower and higher order levels of personality trait hierarchy and growth toward greater maturity [10]. The dimensions of psychopathy might be considered variants of normal personality traits, i.e. with some having more, and others having less psychopathic traits. Given the developmental nature of childhood and adolescence, one might argue that psychopathic traits in children and adolescents are less stable over time as suggested and possibly more changeable than in adulthood.

Research in juveniles published mixed results regarding the stability of psychopathic traits. On the one hand, studies support the idea that psychopathy is relatively stable across adolescence [11–13]. An US-American longitudinal investigation of psychopathic characteristics (callous–unemotional traits, impulsive conduct problems, and narcissism) within a group of aggressive children over an approximately 2 year interval support the notion, that these dimensions are generally stable [11]. One study regarding more than 1500 boys in the public school system showed no age-related fluctuations in reliability, stability and predictive utility of psychopathy across childhood and adolescence suggesting that concern about large changes in personality pathology across childhood and adolescence may be overstated [14]. Psychopathic features in adolescents [15] were found to be moderately to stable in transition from adolescence to adulthood.

On the other hand, assessments of psychopathic traits in a populational cohort of 1631 Canadian children until age 12 identified a decreasing trajectory over time suggesting amenability to change of these traits [16]. Another study examined 370 children from 8 to 14 years old with conduct problems regarding the continuity and change of the three dimensions of psychopathic traits also resulted in the possibility of change, namely that the two other dimensions are more variable than the callous-unemotional dimension [17]. The investigation of a further populational sample of children between ages 7 and 12 (N = 9,78) reported a decreasing developmental trajectory of CU traits in 13.4% of the overall sample and in 37.4% of assessed girls, underlining the need of targeted interventions early in the life-course [18]. Further findings support change and variability of psychopathic traits in adolescence and in transition to adulthood with results from mean- and individual-level analyses revealing a decline of Impulsive Antisociality (e.g. social deviance) from late adolescence to early adulthood [19]. A longitudinal study over a 2–4-year time frame demonstrated a regression to the mean [20]. Identification of psychopathy using the Psychopathic Checklist (PCL) in adolescents was found less reliable over a 2-year period than in adults and increases in psychosocial maturity over time predicted decreases in PCL scores for adolescents [9].

Repeated assessments of changes in Callous/Unemotional, Narcissism, and Impulsivity scores indicate that personality features associated with psychopathy in youth can be reduced through institutional treatment, even in severely behaviorally disordered adolescents (M. F. [21]. Nevertheless, it has to be noted that literature on the different dimensions of psychopathy and its course over time in adolescents is still scarce.

Research focussing more specifically on the underlying dimensions of psychopathic traits demonstrated a moderate level of stability of CU traits over 3 years in a large North-American sample of N = 1216 adolescents who had been arrested for the first time, similar to what has been reported in community samples, as well as an overall decline at older age [22]. A longitudinal study on a twin sample confirmed previous research that genetic factors substantially underlie CU traits during childhood, while non-shared environmental factors have considerable, generally age-specific contributions, over and above genetic factors [23]. A Swedish study investigating the stability of GM traits over 4 years in 1068 adolescents from a community sample reported in summary three profiles with declining levels over time and one profile of adolescents who start with high levels and maintain elevated levels [24]. The time course of II traits in this study suggested three profiles of decreasing levels and a moderate-stable profile [24]. Longitudinal analyses seem to suggest a linkage of attachment to parents with the impulsive-irresponsible psychopathic trait and therefore the influence of environmental factors in the time course of this dimension [25].

The differences of stability of psychopathic traits might partly be explained by the use of different instruments and the large variety of samples as stated by Lynam and colleagues (2007). As alternative measures of psychopathy manifest low rates of agreement in classifying youth as psychopathic even at a single time point [26] there persists the uncertainty if changes of psychopathic scores assessed with different measures reflect instability of psychopathy or measurement errors. The variation of scores on measures of psychopathy might also reflect the method how the components of psychopathy are examined as well as the different developmental stages of the participants [9]. These possible confounding factors were omitted in multiple longitudinal studies on the stability of psychopathic characteristics. One examination of the Psychopathy Checklist: Youth Version (PCL:YV) over a 6-month follow-up period found moderate-to-high stability while the affective factor of the PCL:YV was less stable than the other factors [27]. In a North-American study over a period of approximately 2 years using parent and self-reports (starting sample age approximately 13 years) the rank-order stability of psychopathic traits was high to very high based on parent reports and lower based on self-reports [28]. These findings suggest the stability of psychopathic traits in early adolescence [29]. A longitudinal study in Sweden investigated the stability of psychopathic traits from the approximate age of 13 and onwards for a total of 4 years. The most stable subscales in the yearly assessments using the Youth Psychopathic traits Inventory (YPI) were the impulsive-irresponsible subscale and the grandiose-manipulative subscale indicating that possibly the more behavior-focused dimensions of psychopathic traits are highly-stable across adolescence [24]. A Swedish study on twins examined the importance of genetic and environmental influence for the stability of psychopathic personality between mid- and late adolescence over the course of 3 years measured also using the YPI. Results showed that the three psychopathic personality dimensions were stable at different levels of analysis and linked to a stable higher order general factor (i.e., psychopathic personality factor). Genetic factors contributed substantially to the stability of this general higher order factor, whereas environmental factors were of little importance [30].

In short, current research findings indicate that results regarding the stability of psychopathic traits in adolescents are mixed (e.g. due to differences is assessment, different conceptualization, in treatment or not). The aim of the present study was to add empirical knowledge regarding stability of self-reported psychopathic traits, including the underlying dimensions of GM, CU and II, by studying a sample of at-risk adolescents in Swiss youth welfare and juvenile justice institutions. This knowledge could be informative for treatment approaches and better adherence to therapeutic settings and consecutive improved outcomes in order to prevent recidivism.

## Methods

### Participants

The data used in this study was collected within the MAZ.-project (Swiss Model Project for Clarification and Goal-attainment in Child Welfare and Juvenile-Justice Institutions) (for details see [53]. From 2007 to 2011, an extensive set of computer-based screening questionnaires were administered to 592 children, adolescents and young adults living in 64 different child welfare and juvenile justice institutions in the German-, French-, and Italian-speaking parts of Switzerland at two time points (t1, t2). In parallel, participants were evaluated by their socio-pedagogical caseworkers. Participants were admitted to the institutions by penal law, civil law, or by voluntary placement. Placement by penal law was due to a conviction for a criminal offense (or in some cases being under suspicion of a criminal offense in an ongoing criminal case). Placement by civil law and voluntary placement were due to severe problems in the adolescents´ well-being, behavior or environment. Adolescents were eligible for study participation if they had sufficient linguistic competence in German, French, or Italian and IQ scores above 70. For the current paper, participants aged 12–18 years who had completed the YPI [31] at both time points (t1 and t2) with a time interval of at least 6 months were selected. This yielded a subsample of 162 participants (110 males, 52 females) with an average age of 15.0 (SD = 1.3). Table 1 shows the sample characteristics with respect to age, biological sex, Swiss nationality and type of placement. Male and female participants differed with respect to type of placement (χ^2^ (2, N = 162) = 7.189, p < 0.027), with male participants being underrepresented in civil law placements (59.1% vs. 78.8%), and overrepresented in penal law placements (15.5% vs. 3.8%). There were no differences with respect to age or Swiss nationality.

**Table 1:** Sample characteristics

	n	%	M	SD
Biological sex				
Male	110	67.9		
Female	52	32.1		
Age				
Male			14.9	1.2
Female			15.2	1.4
Nationality				
Swiss	137	84.6		
Non-Swiss	25	15.4		
Placement type				
Civil law	106	65.4		
Penal law	19	11.7		
Voluntary	37	22.8		

### Procedure

Adolescents provided informed consent to participate in the study. Their socio-pedagogical caseworkers had to confirm that they knew the participant well enough to validly answer the study questions. The computerized data collection took place in the institutions and included socio-demographics and information of personal history. In addition, participants and their socio-pedagogical caseworkers completed psychometric screening instruments at t1 and t2, normally at intervals of 1 year. In cases of shorter residence times t2 was collected earlier. Ethical approval for the MAZ.-study was obtained by the Ethics Committee of Basel (Basel-Stadt/City and Basel- Landschaft/Country).

**Table 2:** Mean differences in YPI mean scores and YSR t-scores between t1 and t2

	t1 (SD)	t2 (SD)	p-value
*Total sample (N* = *162)*			
Total mean score	11.05 (1.97)	11.10 (2.28)	0.748
Grandiose-Manipulative	10.15 (2.70)	10.46 (3.02)	0.112
Callous-Unemotional	10.59 (2.08)	10.77 (2.37)	0.313
Impulsive-Irresponsible	12.40 (2.41)	12.06 (2.64)	0.072

### Measures

#### Psychopathic personality traits

Psychopathic personality traits were assessed using a computerized version of the YPI, which is a 50-item self-report questionnaire to assess core personality traits of psychopathy in youth. Each item is scored on a 4-point Likert scale (1 = does not apply at all, to 4 = applies very well). The YPI was designed in line with a three-dimensional conceptualization of psychopathy [1]. The GM dimension or Interpersonal factor includes dishonest charm, manipulation/lying, and grandiosity. The CU dimension or Affective factor includes callousness, unemotionality, and remorselessness. The II dimension or Behavioral factor includes impulsivity, irresponsible behavior, and thrill-seeking. Higher scores reflect higher levels of traits. Assessment was conducted twice (t1, t2) with an interval of at least 6 months. The interval between t1 and t2 varied between 6 and 21 months and was on average 11.1 months (SD = 3.14). Internal consistency based on Cronbach’s alpha at t1 were 0.90 for the YPI total score, 0.89 for GM, 0.70 for CU, 0.77 for II.

### Mental health problems

A computerized version of the Youth Self Report (YSR) [32] was used to measure internalizing and externalizing mental health problems. This questionnaire lists around 120 behavioral and emotional difficulties commonly found in adolescents. Items are scored on a 3-point Likert scale (0 = not true to, 1 = somewhat or sometimes true, 2 = very true or often true). The YSR provides three broadband scales: total problems (TOT), internalizing problems (INT), externalizing problems (EXT). Scores were transformed into t-scores. Internal consistencies within the present sample at t1 were good to excellent (α = 0.93 TOT, α = 0.87 INT, α = 0.86 EXT).

### Measuring reliable change

In order to report essential statements on individual change in psychopathic personality traits analyses were based on the concept of Reliable Change [33, 34]. The Reliable Change Index (RCI) examines whether an individual change is larger than expected due to measurement error of the instruments used. In other words, the difference between the obtained scores is related to the reliability of the measurement. The calculation requires estimates of a scale’s internal consistency or test–retest reliability and the standard deviation at first measurement (SD_Pre_). The threshold for reliable change at a significance level of 0.05 is defined as 1.96 times the standard error of the difference between t1 and t1. Values greater than 1.96 indicate a significant change in the individual. The standard error of the difference (SE_Diff_) is calculated using the formula: $\sqrt {2({\text{SD}}_{{{\text{Pre}}}} \sqrt {1 - \alpha } )^{2} }$.

In the present sample, standard deviations (SD_Pre_) and internal consistency (Cronbach’s α) of scales at t1 provide reasonable estimates of these statistics. Because for the YPI, no satisfying data on the test-retest-reliability in large or comparable samples were found in literature, information on the internal consistency obtained in the present sample was preferred. For the total score, the internal consistency reported below agrees with the test-retest reliability of 0.092 as found among a Canadian nonforensic Sample of Young Adults [35]. For the YPI total score, α was 0.90 and SD_Pre_ of the total mean score was 2.11. A SE_Diff_ of 0.88 and a cut-off for reliable change of 1.73 (0.88 × 1.96) was computed. Thus, any change of > 1.73 in YPI total mean score was considered a reliable change in psychopathic personality traits. On the level of each factor, cut-offs for reliable change were 2.48 for the GM dimension (SD_Pre_ = 2.70, α = 0.89, SE_Diff_ = 1.27), 3.16 for the CU dimension (SD_Pre_ = 2.08, α = 0.70, SE_Diff_ = 1.61), and 3.22 for the II dimension (SD_Pre_ = 2.42, α = 0.77, SE_Diff_ = 1.64).

### Statistical analyses

Statistical analyses were performed using SPSS for Windows, version 27. Differences in YPI mean scores between between t1 and t2 were calculated in paired sample t-tests. To define the groups with reliably increased or decreased YPI scores at t2 cut-offs were set according the aforementioned described RCI. Differences between reliable change groups were calculated using chi-square for categorical variables and ANOVAs for continuous variables. Because of multiple testing in the ANOVAs, Bonferroni correction was applied. Finally, separate exploratory multiple linear regression models were conducted to detect potential influencing factors for YPI total mean scores in total score and each dimension at t2. Participant’s YPI mean scores at t1, age, biological sex, time span between t1 and t2 and behavior measures at t1 were included as independent variables. Significance levels for all analyses were set at α = 0.05.

## Results

First, psychopathic trait scores of the YPI between t1 and t2 were compared at group level. Results showed no significant differences on the total score as well as on the underlying dimensions (Table 2).

**Fig. 1:**
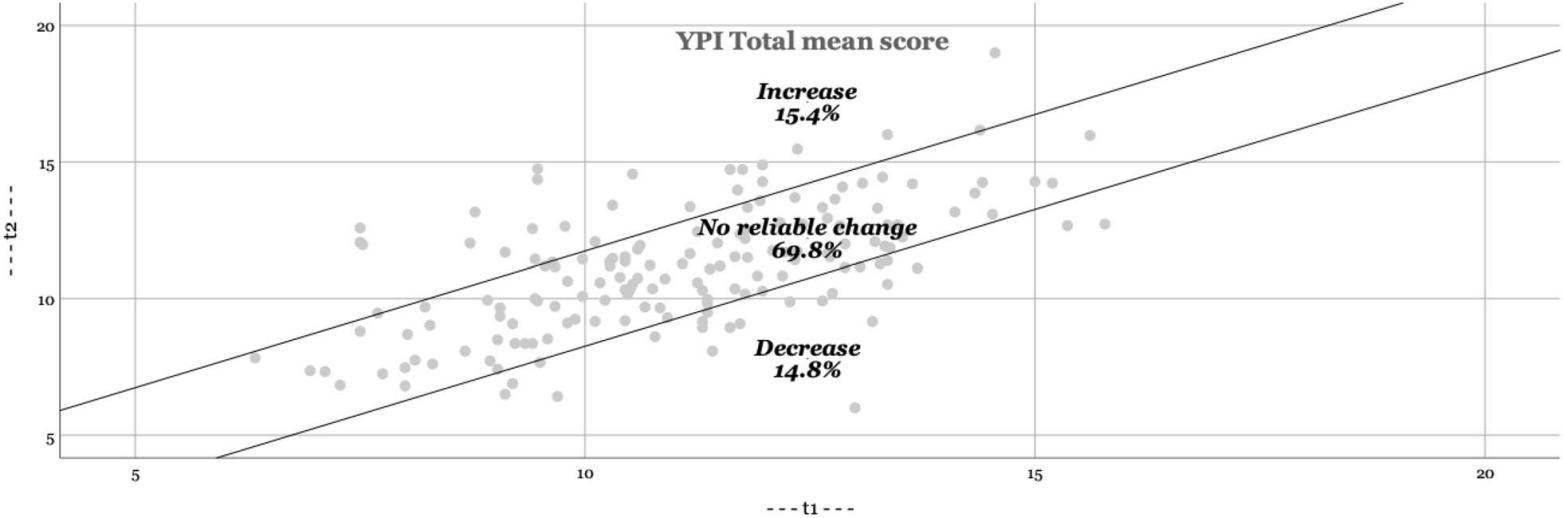
Reliable changes YPI Total score

**Fig. 2:**
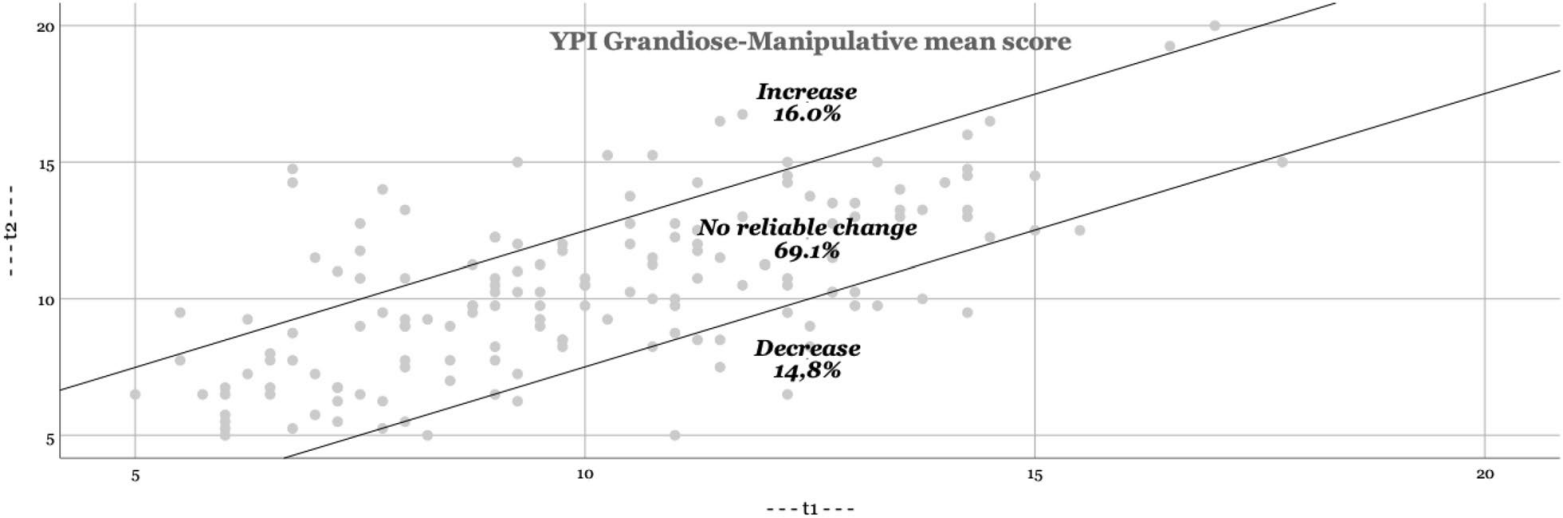
Reliable changes GM traits

**Fig. 3:**
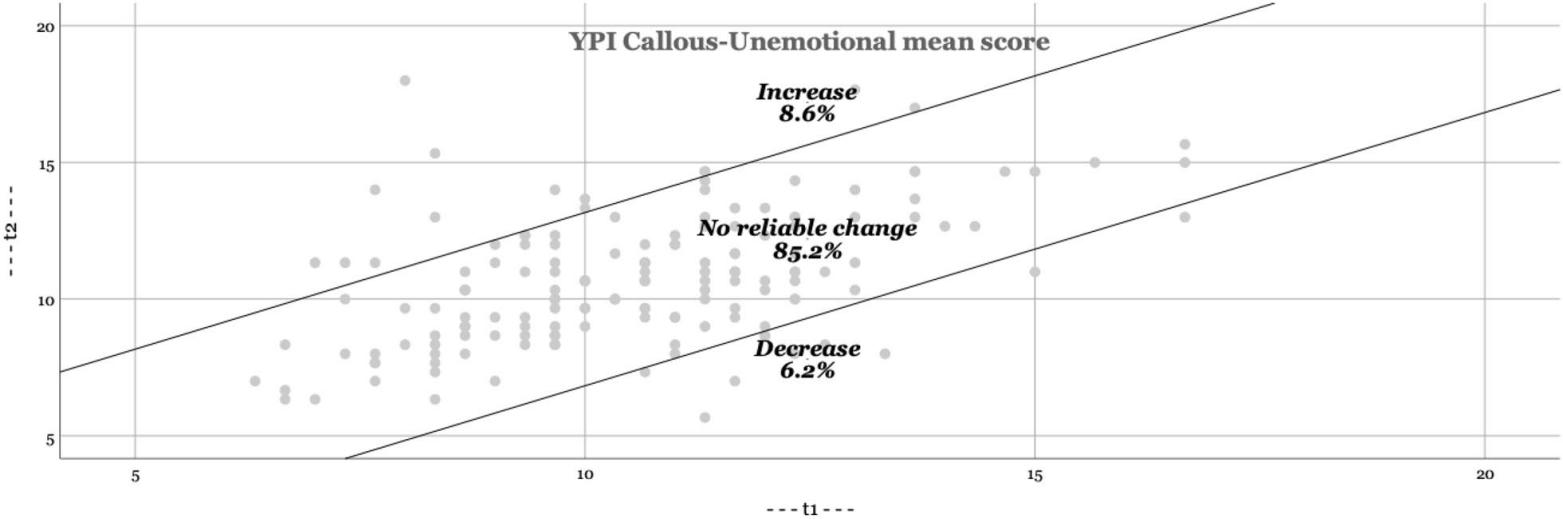
Reliable changes CU traits

**Fig. 4:**
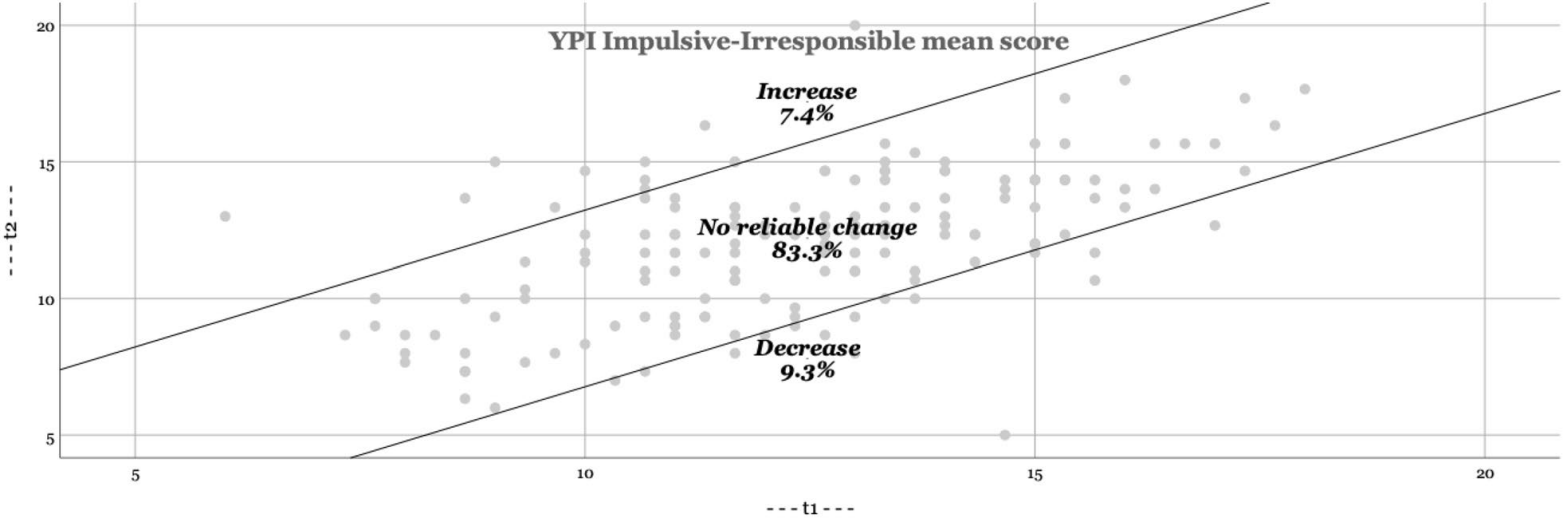
Reliable changes II traits

In Figs. 1, 2, 3, 4, the RCI are presented visually. The majority of adolescents do not change significantly (YPI total score and GM dimension: approximately 70; CU and II dimension: approximately 85%). The percentage that improves or deteriorates is overall relatively evenly distributed for both the YPI total score and the underlying dimensions.

In Table 3 various predictive linear regressions models for the YPI total score and the three underlying dimensions at t2 are presented. At step 1, only the equivalent at t1 of the outcome of the model is included (e.g., the YPI total score at t1 as a predictor of the YPI total score at t2). At step 2, age, biological sex and time between t1 and t2 are added. Finally, emotional problems and behavioral problems are added to the model (Step 3). In all models, the equivalent at t1 of the outcome is the strongest predictor. The R^2^ does not increase noticeably, except for CU-traits (Step 1: 25.2%, Step 3: 31.6%). In addition to the YPI total score at t1, biological sex is of predictive value for the YPI total score at t2 in the final model (step 3). Boys have significantly higher YPI total scores at t2 than girls. We also see this biological sex distinction in the final CU-traits model. A third significant predictor in the final CU-traits model, next to CU-traits at t1 and biological sex, is emotional problems (YSR INT): the more emotional problems at t1, the fewer CU-traits at t2. Finally, behavioral problems (YSR EXT) were found to be predictive of II-traits at t2 (over and above II-traits at t1), with higher behavioral problems scores predicting higher II scores.

**Table 3:** Linear regressions predicting YPI Total mean score and YPI dimensions at t2

*Step*	*Predictor*	Unstandardized coefficients		Standardized coefficients		*R*^*2*^	*F*
		*B*	*SE*	*β*	*p*		
YPI total mean score at t2							
1						0.360	91.377
	YPI Total mean score at t1	0.697	0.073	0.603	< 0.001		
2						0.374	25.043
	YPI Total mean score at t1	0.644	0.076	0.557	< 0.001		
	Age	0.173	0.116	0.096	0.138		
	Biological sex (1 = male; 2 = female)	− 0.703	0.317	− 0.144	0.028		
	Time span in months (t1-t2)	− 0.021	0.047	− 0.028	0.663		
3						0.383	17.625
	YPI Total mean score at t1	0.576	0.088	0.498	< 0.001		
	Age	0.169	0.115	0.094	0.146		
	Biological sex (1 = male; 2 = female)	− 0.718	0.321	− 0.147	0.027		
	Time span in months (t1-t2)	− 0.009	0.047	− 0.012	0.853		
	YSR INT at t1	− 0.026	0.017	− 0.105	0.129		
	YSR EXT at t1	0.037	0.020	0.146	0.069		
YPI total Grandiose-Manipulative mean score at t2							
1						0.414	114.873
	YPI Grandiose-Manipulative mean score at t1	0.725	0.068	0.646	< 0.001		
2						0.424	30.579
	YPI Grandiose-Manipulative mean score at t1	0.681	0.070	0.608	< 0.001		
	Age	0.182	0.146	0.077	0.216		
	Biological sex (1 = male; 2 = female)	− 0.711	0.403	− 0.110	0.080		
	Time span in months (t1-t2)	− 0.065	0.060	− 0.068	0.281		
3						0.424	20.754
	YPI Grandiose-Manipulative mean score at t1	0.650	0.077	0.580	< 0.001		
	Age	0.170	0.147	0.072	0.249		
	Biological sex (1 = male; 2 = female)	− 0.714	0.410	− 0.111	0.084		
	Time span in months (t1-t2)	− 0.053	0.061	− 0.055	0.384		
	YSR INT at t1	− 0.021	0.22	− 0.065	0.329		
	YSR EXT at t1	0.033	0.024	0.098	0.176		
YPI total Callous-Unemotional mean score at t2							
1						0.252	55.178
	YPI Callous Unemotional mean score at t1	0.578	0.078	0.506	< 0.001		
2						0.300	18.225
	YPI Callous Unemotional mean score at t1	0.515	0.080	0.451	< 0.001		
	Age	0.166	0.127	0.089	0.192		
	Biological sex (1 = male; 2 = female)	− 1.187	0.352	− 0.234	< 0.001		
	Time span in months (t1-t2)	0.053	0.052	0.070	0.314		
3						0.316	13.384
	YPI Callous Unemotional mean score at t1	0.475	0.083	0.416	< 0.001		
	Age	0.168	0.126	0.090	0.185		
	Biological sex (1 = male; 2 = female)	− 1.144	0.351	− 0.226	0.001		
	Time span in months (t1-t2)	0.069	0.052	0.091	0.188		
	YSR INT at t1	− 0.042	0.019	− 0.164	0.026		
	YSR EXT at t1	0.034	0.020	0.128	0.098		
YPI total Impulsive-Irresponsible mean score at t2							
1						0.320	76.778
	YPI Impulsive-Irresponsible mean score at t1	0.624	0.071	0.569	< 0.001		
2						0.326	20.429
	YPI Impulsive-Irresponsible mean score at t1	0.595	0.072	0.543	< 0.001		
	Age	0.192	0.140	0.093	0.172		
	Biological sex (1 = male; 2 = female)	− 0.335	0.370	− 0.059	0.366		
	Time span in months (t1-t2)	− 0.063	0.056	− 0.075	0.266		
3						0.339	14.740
	YPI Impulsive-Irresponsible mean score at t1	0.499	0.085	0.456	< 0.001		
	Age	0.188	0.139	0.091	0.178		
	Biological sex (1-male; 2 = female)	− 0.337	0.370	− 0.060	0.364		
	Time span in months (t1-t2)	− 0.044	0.057	− 0.053	0.435		
	YSR INT at t1	− 0.018	0.020	− 0.064	0.368		
	YSR EXT at t1	0.056	0.025	0.188	0.025		

*SE* standard error of B. Biological sex: 1 male, 2 female, *Time span (t1-t2)* time interval between t1 and t2 in months, *YSR INT* Internalizing problems (T-score), *YSR EXT* Externalizing problems (*T*-score)

Finally, the three reliable change groups (increasers, no reliable change, decreasers) were compared on several relevant variables (Table 4). Only the reason for placement significantly differed between the three groups when taken the YPI Total score as the outcome into account. Adolescents placed voluntarily were more often found to show no reliable change, adolescents placed under penal law were more often in the group that clinically significantly improved.

**Table 4:** Differences between reliable change groups

	Increase *(%/M, SD)*	No reliable change *(%/M, SD)*	Decrease *(%/M, SD)*	*Chi*^*2*^*/F*	*p*
YPI Total mean score					
Biological sex (m, f)	18.2, 9.6	67.3, 75.0	14.5, 15.4	1.998	0.368
Age	14.5, 1.2	14.6, 1.3	14.3, 1.4	0.586	0.558
Time span in months (t1–t2)	11.6, 3.6	11.0, 3.0	11.2, 3.6	0.402	0.670
Nationality (Swiss, Non-Swiss)	16.0, 15.3	72.0, 69.3	12.0, 15.3	0.186^a^	0.991
Placement type (civil law, penal law, voluntary)	18.9, 10.5, 8.1	66.0, 57.9, 86.5	15.1, 31.6, 5.4	10.292^a^	0.036
YSR INT at t1	57.2, 9.4	59.0, 9.3	61.8, 8.4	1.597	0.206
YSR EXT at t1	61.2, 8.9	60.5, 8.6	62.8, 8.9	0.683	0.507
YPI Grandiose-Manipulative mean score					
Biological sex (m, f)	17.3, 13.5	68.2, 71.2	14.5, 15.4	0.381	0.826
Age	14.6, 1.2	14.6, 1.3	14.3, 1.4	0.378	0.686
Time span in months (t1–t2)	11.5, 3.1	11.0, 3.1	10.9, 3.6	0.311	0.733
Nationality (Swiss, Non-Swiss)	20.0, 15.3	68.0, 69.3	12.0, 15.3	0.451^a^	0.798
Placement type (civil law, penal law, voluntary)	17.9, 10.5, 13.5	68.9, 57.9, 75.7	13.2, 31.6, 10.8	5.508^a^	0.239
YSR INT at t1	58.0, 9.5	58.8, 9.5	61.5, 7.2	0.999	0.370
YSR EXT at t1	61.7, 11.0	59.9, 8.2	64.7, 9.0	2.982	0.054
YPI Callous-Unemotional mean score					
Biological sex (m, f)	10.2, 5.8	84.5, 86.5	5.5, 7.7	1.034^a^	0.596
Age	14.4, 1.6	14.6, 1.3	14.7, 1.3	0.224	0.799
Time span in months (t1–t2)	10.7, 3.2	11.0, 3.1	12.2, 3.9	1.040	0.356
Nationality (Swiss, Non-Swiss)	12.0, 8.0	88.0, 84.7	0.0, 7.3	2.238^a^	0.327
Placement type (civil law, penal law, voluntary)	10.4, 5.3, 5.4	83.0, 89.5, 89.2	6.6, 5.3, 5.4	1.331^b^	0.856
YSR INT at t1	59.1, 9.6	58.7, 9.3	64.1, 7.3	1.587	0.208
YSR EXT at t1	60.8, 11.3	60.9, 8.7	61.6, 8.9	0.032	0.969
YPI Impulsive-Irresponsible mean score					
Biological sex (m, f)	10.0, 1.9	80.0, 90.4	10.0, 7.7	3.770^a^	0.152
Age	14.8, 1.2	14.5, 1.3	14.7, 1.2	0.268	0.765
Time span in months (t1–t2)	12.9, 3.1	10.9, 3.0	11.2, 3.9	3.001	0.053
Nationality (Swiss, Non-Swiss)	12.0, 6.6	76.0, 84.7	12.0, 8.8	1.272^a^	0.529
Placement type (civil law, penal law, voluntary)	7.5, 10.5, 5.4	84.9, 63.2, 89.2	7.5, 26.3, 5.4	8.463^b^	0.076
YSR INT at t1	55.5, 10.2	59.4, 9.3	59.5, 7.4	0.985	0.376
YSR EXT at t1	59.7, 9.3	61.2, 9.1	59.6, 7.1	0.338	0.714

*YSR INT* Internalizing problems (T-score), *YSR EXT* Externalizing problems (*T*-score)

^a^2 cells have an expected frequency less than 5

^b^4 cells have an expected frequency less than 5

## Discussion

The aim of the present study was to add empirical knowledge regarding the stability of self-reported psychopathic traits, including the underlying dimensions of GM, CU and II, in at-risk youths. Based on our results psychopathic traits seem to be stable at group level over an average follow-up time of 11 months. In addition, we found similar results on an individual level (as based on the RCI); the majority of adolescents did not show a clinically significant change. This is in line with previous findings using external assessments [27, 36]. Still, on a total score level of the YPI we observed a reliable decrease of scores in 14.8% and about the same amount of increase of the sample. Given the relatively short time span, the life period of adolescence (vs earlier childhood) and the institutional nature of the sample this amount may not be expected. A possible explanation is, that the trajectories of GM and II show more heterogeneity than the evolution of CU in childhood, which may as well be assumed for adolescence. While GM and II decline on average in childhood from 8 to 14 years [17] this may still be applicable for the subsequent developmental period of adolescence and therefore result in an overall higher malleability as previously thought. Possibly the use of different instruments (self-report vs external assessment) might pretend a decrease in our sample which would not be substantiated in a third party instrument. A further point might be, that the placement in institutions removed the adolescents in our sample of an overly dysfunctional parental or unfavourable psychosocial setting which benefitted a proportion of the assessed youth in regard to a decrease of II and GM traits. In line with the reasoning of different variability of the three assessed dimensions, we could reproduce the findings of earlier studies suggesting that the CU dimension is less amenable to change than the other dimensions II and GM [17]. The amount of change of the CU traits in our sample (6.2% decrease) was substantially lower than findings in a large populational sample of children between ages 7 and 12 (decreasing developmental trajectory of CU traits in 13.4%) [18]. This may not be surprising given the background of our sample and the general lower malleability of the CU trait.

Regarding predictor factors for psychopathic traits, we found that psychopathic traits at t1 were the strongest predictor for psychopathic traits at t2. This did not only account for the YPI total score, but also for all underlying dimension. Over and above the psychopathic trait scores at t1, biological sex was also found to be a significant predictor for the YPI total score as well as CU scores; adolescent males had higher psychopathic trait/CU trait scores at t2 compared to adolescent females. This is in line with literature reporting higher psychopathic traits in adult males in comparison to female sex [37] which also supports the notion of stability of psychopathic traits from adolescents to adulthood. Furthermore, internalizing mental health problems—which often manifest as symptoms of anxiety and depression—were negatively associated with higher levels of CU traits at t2. This is in line with the concept of the primary variant subgroup of youth with CU traits which is associated with low levels of anxiety, trauma, and Posttraumatic Stress Disorder (PTSD) [38]. Current knowledge regarding the dimensions of psychopathy is, that the interpersonal facet is associated with a lack of internalizing symptoms and somewhat enhanced proneness to externalizing problems [39]. Finally, externalizing mental health problems were positively related to II traits at t2 over and above II traits at t1. This is in line with other studies [40]. This connection might warrant further attention as other studies linked elevated externalizing problems like (especially psychological) child-to-parent violence to high levels of II traits [41]. Overall we could confirm the reported association of impulsive-irresponsible facet with elevations disinhibitory externalizing symptomatology [39].

One of the main finding of this study points to the stability of psychopathic traits in adolescence while assessed by self-report. The resulting implications have several facets which warrant attention. The stability of psychopathic traits in adolescence is reflected in neurobiological features. Respective literature on the neurobiology of psychopathic traits in youth suggests a decreased empathic response to distress principally associated with a reduced amygdala response to fear, sadness or pain of others and an impairment in the ventromedial prefrontal cortex and caudate tied with dysfunctions in reinforcement-based decision making [42]. It is speculated about a genetic and hence heritable contribution to these structural and functional brain abnormalities [43]. As well, the extend of environmental influences on the development of the amygdala, ventromedial prefrontal cortex and caudate is still debated [42]. Findings in personality disorders suggest predisposing genetic loci or subsequent epigenetic changes through environmental influences (i.e. childhood maltreatment) that promote certain personality traits [44]. As a consequence, psychopathic traits could as well be viewed as a neurodevelopmental stress-related disorder [43].

Psychosocial impairments in adolescents are consistently reported due to psychopathic traits and include lower global Assessment of Functioning scores and higher rates of school drop-outs [45] and even intergenerational transmission of psychopathic traits thorough psychosocial risk factors (e.g. employment and accommodation problems) is reported [46]. These prolonged psychosocial effects seem to be correlated with concurrent (and hence possible stable) psychopathic traits.

From a forensic point of view, the link between violent and nonviolent criminality and psychopathic traits is robustly reproduced in literature regarding adults as well youths [45–48]. The stability observed in psychopathic traits in this study could imply that maladaptive symptoms of psychopathic traits are not temporary as well, and hints at the possible benefit of diagnosing and assessing young people with potential (or present) risk of criminal behaviour.

From a wider clinical point of view, well observable aspects of psychopathic traits in youths include Oppositional Defiant Disorder and Conduct Disorder based on the DSM-V. The aforementioned emotion recognition impairments in connection with higher levels of impulsivity and narcissism seem to be associated with maladaptive behaviour in clinical settings [49]. The implication of variability of a minor proportion of affected youth point to the need of developing early tailored interventions to positively affect still malleable traits or compensate stable impairments.

With regard to the RCI outcomes, we generally did not find any differences between the three groups (i.e. increasers, no reliable change, decreasers) except for reason of placement for the YPI total score. This seemed to concern in particular the overrepresentation of the group of voluntarily placed adolescents in the no reliable change group and to a lesser extent the overrepresentation of penal placed adolescents in the decreasers group. The latter group is particularly interesting because it may provide more information about the treatment options of young offenders in order to reduce psychopathological traits. Unfortunately, based on our data it was not possible to investigate this in more detail. However, data of a 2-year follow-up on juvenile offenders with high scores on the PCL:YV suggested that intensive treatment was associated with relatively slower and lower rates of serious recidivism, even after controlling for the effects of non-random assignment to treatment groups and release status [50]. In addition, a recent study on male detained youth aged 14 to 18 years old from Portugal showed promising results in an intervention group treated with a specialized program with moderate to large decreases in psychopathic traits on global and factor level at post-treatment and 6-months follow-up [51]. This notion is supported by our data as all adolescents were placed in institutions with pedagogical concepts with considerable effort to better socialize these youths. Nevertheless, just as many adolescents increased as decreased in their psychopathic traits, while the majority remained stable. This strongly suggests that there is indeed a need for specialized programs and treatment as usual seems to contribute hardly when it comes to attenuating psychopathic traits. This points to possible beneficial effects of an intensive and structured therapeutic setting as described for adults with high levels of psychopathic traits [52].

## Limitations

The results of the current study have to be seen in the light of various limitations: First, as reported in the literature, short time spans between assessments tend to have higher stability than longer time spans [24]. With a mean time period of 11 months this observation has to be kept in mind before qualifying the stability of longer term psychopathic traits in adolescents. Second, is the modality of the assessment, i.e. self-reports on psychopathic traits. While being a more ecological method, external assessments through parent and teacher ratings are more objective. Third, the heterogeneity of our sample calls for subgroup analyses. However, the size of our sample often does not allow for this. The sample size limitation is explainable by the characteristics of the sample (i.e. adolescents hard to recruit and reach) and the study design (medium-term longitudinal study, a very extensive test battery to better understand the strengths and difficulties within this very complex group) in the field of residential youth care with at-risk (for an overview of the study design, see Schmid et al. [53]. Fourth, substance misuse was not explicitly assessed in the study, a factor consistently associated with psychopathic traits and the persistence of antisocial behaviour [43, 54]. Finally, the youth care system in Switzerland is unique, as youth with civil and criminal law decision can be placed in the same institutions (for more details, see Jäggi et al. [55]. Consequently, our results are not easily generalizable to other countries and legal systems. Hence, more research in other countries is needed.

## Implications

The considerable stability of psychopathic traits in adolescents is linked to the clinical worry of subsequent criminal and self-harming (e.g. substance use) behaviour [56]. With the persistence of psychopathic traits into adulthood, psychosocial impairments are documented as well (e.g. work functioning, relationships or education) [43, 45, 57]. Psychopathy is known as a strong predictor of chronic offending trajectories from early adolescence to adulthood and is also associated with longer periods of imprisonment [58]. Incarcerated youth within the criminal justice system are affected by complex health problems, health-risk behaviours, and high rates of premature death [59] together with the aforementioned substance use and sensation seeking behaviour can result in high morbidity and mortality rates. Of note, psychopathy is often comorbid with other psychiatric disorders and increases the risk of physical health problems and accidents [43].

Besides the negative consequences of psychopathy on the individual, violence associated with psychopathic traits constitutes a substantial portion of the societal burden [60]. As prevention of violence is a major goal of public health systems, psychopathic traits merit attention of public health interventions and is therefore a public health issue. Consequently, it is important to identify children and adolescents at-risk of developing and increasing psychopathic traits which could be screened using self-report tools. Once identified, the need arises to intervene in the early life course in psychopathic traits and not assume that these traits will disappear on over time. The recommended treatment modality for reducing childhood conduct problems in children and adolescents at risk of psychopathy is parent management training in meta-analyses, with treatment gains that are maintained over 3 or more years after the intervention [61]. Further, for adolescence the treatment method of multisystemic therapy was reported as effective [64]. As inconsistent, conflict-ridden and harsh parenting and child maltreatment are important risk factors for the development of psychopathy [43], the integration of parents into the therapy of children and adolescents at-risk of psychopathy would probably benefit the treatment outcome. Evidence of efficacy was found for further psychosocial treatments for conduct problems including problem-solving skills training, anger control and social skills training, contingency management, cognitive–behavioural interventions, family therapy and multisystemic therapy [62, 63]. Parts of these interventions are often established in institutions with a penal background, which might explain our results regarding the trend of decreasers in YPI total score in penal institutions. In addition, in order to better understand our results, more research is warranted. This research should focus on the use of third party assessment and expert opinions/clinical judgements in addition to the use of self-report. A longer follow-up time is also recommended. Furthermore, more in depth research in gender differences (which was not possible in the current study due to the limited numbers of girls in our sample) as well as the influence of substance use problems on the stability of psychopathic traits might be of interest. Finally, given the aforementioned unique situation in Switzerland, it is important that our results be replicated in other countries, other legal systems, and other modalities (such as juvenile justice institutions, forensic psychiatry, outpatient care).

## Conclusions

Our results showed that self-reported psychopathic traits in adolescents in residential care over a mean time interval of 11 months were relatively stable. The question arises if this stability can also be found over longer time periods as well as with other assessment modalities, such as expert opinions/clinical judgements (using e.g. the PCL:YV). Hence, additional research is needed to better understand our results.

## Data Availability

The datasets generated and/or analysed during the current study are not publicly available due [property of the federal ministry of justice] but are available from the corresponding author on reasonable request.

## Abbreviations

YPI: Youth Psychopathic traits Inventory
GM: Grandiose-Manipulative dimension of psychopathy
CU: Callous-Unemotional dimension of psychopathy
II: Impulsive-Irresponsible dimension of psychopathy
YSR: Youth Self Report
TOT: Total problems
INT: Internalizing problems
EXT: Externalizing problems
PCLYV: Psychopathic Checklist (PCL): Youth Version
MAZ: Modellversuchs Abklärung und Zielerreichung in stationären Massnahmen (English translation: Swiss Model Project for Clarification and Goal-attainment in Child Welfare and Juvenile-Justice Institutions)
